# Sampling and Kriging Spatial Means: Efficiency and Conditions

**DOI:** 10.3390/s90705224

**Published:** 2009-07-02

**Authors:** Jin-Feng Wang, Lian-Fa Li, George Christakos

**Affiliations:** 1 Institute of Geographic Sciences & Nature Resources Research, Chinese Academy of Sciences, Beijing, China; E-Mail: lilf@lreis.ac.cn; 2 Department of Geography, San Diego State University, San Diego, CA, USA; E-Mail: gchrista@mail.sdsu.edu

**Keywords:** random field, mean Kriging, spatial dependence, GIS

## Abstract

Sampling and estimation of geographical attributes that vary across space (e.g., area temperature, urban pollution level, provincial cultivated land, regional population mortality and state agricultural production) are common yet important constituents of many real-world applications. Spatial attribute estimation and the associated accuracy depend on the available sampling design and statistical inference modelling. In the present work, our concern is areal attribute estimation, in which the spatial sampling and Kriging means are compared in terms of mean values, variances of mean values, comparative efficiencies and underlying conditions. Both the theoretical analysis and the empirical study show that the mean Kriging technique outperforms other commonly-used techniques. Estimation techniques that account for spatial correlation (dependence) are more efficient than those that do not, whereas the comparative efficiencies of the various methods change with surface features. The mean Kriging technique can be applied to other spatially distributed attributes, as well.

## 1. Introduction

Spatial estimation techniques have many applications in the study of attributes, such as soil and land cultivation properties, water resource parameters, air pollution variables, population disease characteristics, regional poverty levels and agricultural production indices [1–3]. In addition, the assessment of the uncertainty associated with the generated estimates is as important as attribute estimation itself. E.g., if the accuracy of an attribute estimate is low (i.e., the uncertainty is high), the estimate is rather useless or even misleading. If, on the other hand, the accuracy is high, the estimated attribute value could be used in decision-making, such as the international negotiations of carbon emission reduction to address the global warming challenge.

In the GIS context, there are two main methodologies of area mean attribute estimation:

- One general methodology focuses on *spatial statistics*-based estimates using a set of observations/measurements across space [4–7]. This methodology includes objective analysis-, superpopulation model-, sampling- and design-based techniques [7–12].
- Another major methodology relies on *physical mechanism*-based estimates in addition to the datasets available [13–17]. This methodology includes Bayesian maximum entropy (BME) techniques [18–21], variational assimilation and Kalman techniques [22–26].

In theory, methodology *b* is superior to methodology *a* since, in addition to the available datasets, it can offer a more physically meaningful and informative analysis of the phenomenon of interest by accounting for valuable knowledge in the form of scientific theories, physical laws and primitive equations [27–29]. In practice, however, this kind of knowledge is often not available (or, if available, the computational procedures to account for it do not yet exist or are of limited use), in which case the efficiency of the techniques belonging to methodology *a* proves to be very useful. It is for this reason that a spatial statistics-based technique belonging to methodology *a* is considered in this work.

Spatial statistics-based techniques seek to account for uncertainty caused by gaps between the sampled sites [30]. A simple sample mean is an unbiased estimate of both the observable population and the superpopulation means, under the conditions of a second-order stationary object surface and a randomly distributed sample over space [31,32], but the variance of the estimate is not minimized. Spatial sampling techniques improve the efficiency of sampling and estimation by taking spatial correlation (dependence) into account [33], but that does not always guarantee that the estimation variance is minimized. Kriging leads to an unbiased estimate for unsampled values with the least variance [7], but the estimation of the mean attribute in terms of a summation of individual estimates at unsampled sites may also accumulate the errors of each individual estimate. Kriging the attribute mean across space yields an estimation of the area mean that is unbiased and has the minimum estimation variance. The technique has already existed in the literature for several decades – Kriging was originally developed in the context of Wiener-Kolmogorov estimation and objective analysis [6,10,34–36]. In the present work, our concern is twofold: the estimation of the spatial attribute mean over a specified area using the mean Kriging technique, and the study of the probability distribution of these estimates over the area of interest. Practical insight is gained in terms of a temperature dataset and a land use dataset distributed in space, in which the mean Kriging analysis is compared with previous techniques, such as ordinary Kriging, spatial random sampling and simple random sampling techniques.

## 2. Spatial Random Field Representation of Attributes and Their Means

Let a geographical attribute be represented mathematically by the *spatial random field* (SRF), *Y*(***s***) in the sense of Christakos [34]. The ***s*** denotes the spatial coordinates of location ***s*** and the SRF includes a family of spatially correlated (geographically dependent) random variables *y*_1_,…,*y_n_* at sample points ***s***_1_,…,***s****_n_*. A number of concepts of GIS interest can be defined in the SRF context, see below.

The *observed spatial population mean* (OSPM) over an area ℜ of the attribute represented by the SRF *Y*(***s***), also called the *observed area mean*, is defined as:

(1) $${\bar{Y}}_{ℜ}=\frac{1}{ℜ}\int_{ℜ}Y(s)ds$$

where ***s*** varies within ℜ. The *Ȳ*_ℜ_ is a random quantity, i.e., even when considering the same area ℜ, one may get different results if the *Ȳ*_ℜ_ is computed over different realizations.

In the GIS context, the *superpopulation mean* (SPM) of the SRF at each location ***s***, also called the *stochastic mean*, is defined as:

(2) $$m(s)=E[Y(s)]=\int ds\;\psi (s)\;f_{Y}\;(s)$$

where *E*[·] denotes stochastic expectation, the *f*_Y_(***s***) is the probability density function (pdf) of the SRF *Y*(***s***) and *ψ*(***s***) is the SRF realization at ***s***. The *m*(***s***) is the average value of all SRF realizations at each ***s*** and is a non-random quantity. Note that it has to a single value *m* for all locations ***s***, as long as the SRF is 1^st^-order stationary, i.e., *E*[*Y*(***s***)] = *const*. for all ***s***.

The *simple sample mean* (SSM) is defined as:

(3) $${\bar{Y}}_{n}=\frac{1}{n}\sum_{i=1}^{n}y_{i}$$

where *y_i_* are the corresponding random variables at locations ***s****_i_* (*i* = 1,…,*n*) within the study area ℜ. The *Ȳ_n_* is a random quantity, since the random variables *y_i_* can assume various values (realizations) and the *n* sample units can be drawn randomly across space. Equation (3) would be the best linear unbiased estimate of both the observable population mean and the superpopulation mean if the ***s****_i_* (*i* = 1,…,*n*) are randomly distributed over space and the corresponding SRF is 1^st^-order spatial stationary; i.e., $E\left[{\bar{Y}}_{n}\right]=\frac{1}{n}\sum_{i=1}^{n}E\left[y_{i}\right]=\frac{1}{n}\mathrm{nm}=m$.

The *weighted sample mean* (WSM) is defined as:

(4) $${\bar{Y}}_{n}^{w}=\sum_{i=1}^{n}w_{i}\;y_{i}$$

where *w_i_* are weights assigned to the random variables *y_i_* (*i* = 1,…,*n*). Again, ${\bar{Y}}_{n}^{w}$ is a random quantity. Clearly, *Ȳ_n_* is a special case of ${\bar{Y}}_{n}^{w}$ when all weights are equal, i.e., $w_{i}=\frac{1}{n}$.

## 3. SSM of OSPM

The variance of SSM is given by:

$$\sigma_{{\bar{Y}}_{n}}^{2}=E{\left[{\bar{Y}}_{n}-{\bar{Y}}_{ℜ}\right]}^{2}=E{\left[\frac{1}{n}\sum_{i=1}^{n}\;y_{i}-\frac{1}{ℜ}\int_{ℜ}ds\;Y(s)\right]}^{2}=\sigma_{p}^{2}\;F(n)$$

where $\sigma_{p}^{2}$ is dispersion variance of the population of the target area and *F*(*n*) is a variance reduction factor and estimated by [37]:

(5) $$\{\begin{array}{cc} F(n)=1/n\; & \text{simple random sampling}\\ F(n)=\left\{1-E\left[r\left(s_{i}-s_{j}\right)|ℜ\right]\right\}/n\; & \text{spatial random sampling}\\ F(n)=\left\{1-E\left[r\left(s_{i}-s_{j}\right)|(ℜ/k)\right]\right\}/n & \text{spatial stratified sampling} \end{array}$$

where *n* is the number of sampling units and *k* is the number of strata; simple random sampling disregards spatial correlation, whereas the spatial random sampling and spatial stratified sampling take spatial correlation into account; the *r*(*s_i_ – s_j_*) expresses spatial dependence between any two sites *s_i_* and *s_j_*; E[*r*(*s_i_ – s_j_* |·] is usually a positive quantity lying in the interval [0, 1] and can be estimated directly from the observed *r*(*s_i_ – s_j_*) values and the probability distribution of distances over the study area ℜ or the strata ℜ/*k* [38].

Next we investigate the role of spatial correlation and sampling design on the sample mean variance. Let *n_0_*, *n_r_*, and *n_s_* denote the numbers of sample units for simple random sampling, spatial random sampling and spatial stratified sampling, respectively. To assure the required estimation accuracy $\sigma_{{\bar{Y}}_{n}}^{2}$, one finds from (5) that:

(6) $$\frac{n_{r}}{n_{0}}=1-E\left[r\left(s_{i}-s_{j}\right)|ℜ\right]$$

(7) $$\frac{n_{s}}{n_{0}}=1-E\left[r\left(s_{i}-s_{j}\right)|\left(ℜ/k\right)\right]$$

(8) $$\frac{n_{s}}{n_{r}}=\frac{1-E\left[r\left(s_{i}-s_{j}\right)|\left(ℜ/k\right)\right]}{1-E\left[r\left(s_{i}-s_{j}\right)|ℜ\right]}$$

Because 0 ≤ *E*[*r*(*s_i_* − *s_j_*|ℜ] ≤ *E*[*r*(*s_i_* − *s_j_*|ℜ/*k*] by Tobler’s first law of geography [39], which argues that nearby attribute values are more similar than those that are further apart; consequently, *n_s_* ≤ *n_r_* ≤ *n_0_* [from Equations (6)–(8)]. Similarly, given the same sample size *n*, one can compare the variances of the three sampling mean estimates and conclude that: Var (simple random sampling mean) ≥ Var (spatial random sampling mean) ≥ Var (spatial stratified sampling mean). The conclusion is that the stratified sampling is generally more efficient in reducing estimation variance than random sampling, and the sampling regarding spatial correlation is generally more efficient than that which neglects spatial autocorrelation. Efficiency refers to the fact that using fewer sample units leads to higher estimation accuracy. The SSM property of best linear unbiased estimation when sampling 1^st^-order spatial stationary SRF would not be retained when sampling 2^nd^-order stationary SRF, a drawback that can be overcome by WSM or mean Kriging.

## 4. Mean Kriging of OSPM

One can estimate the OSPM (*Ȳ*_ℜ_) by the WSM ( ${\bar{Y}}_{N}^{w}$) using a Kriging technique (a presentation of the various Kriging techniques and their relation to other spatial estimation methods can be found in [34]). The WSM ${\bar{Y}}_{n}^{w}$ satisfies two conditions: (*a*) it is an unbiased estimate of the OSPM *Ȳ*_ℜ_, and (*b*) it minimizes the mean squared estimation error. Condition (*a*) implies that:

$$E\left[{\bar{Y}}_{n}^{w}\right]=E\left[{\bar{Y}}_{ℜ}\right]\;\;\text{or}\;\;E\left[\sum_{i=1}^{n}w_{i}\;y_{i}\right]=E\left[\frac{1}{ℜ}\int_{ℜ}dsY(s)\right]$$

Since the SRF *Y*(***s***) is 1^st^ order spatial stationary *E*[*y_i_*] = *E*[*Y*(***s***)], that leads to:

$$\sum_{i=1}^{n}w_{i}=1\;$$

The mean squared estimation variance is given by:

$$\begin{array}{ll} \sigma_{{\bar{Y}}_{n}^{w}}^{2} & =E{\left[{\bar{Y}}_{n}^{w}-{\bar{Y}}_{ℜ}\right]}^{2}=E{\left[\sum_{i=1}^{n}w_{i}\;y_{i}-\frac{1}{ℜ}\int_{ℜ}dsY(s)\right]}^{2}\\ & =\frac{1}{{ℜ}^{2}}\int_{ℜ}\int_{ℜ}ds\;ds^{\prime }\;c_{Y}\;\left(s,s^{\prime }\right)\;+\sum_{i=1}^{n}\sum_{j=1}^{n}w_{i}\;w_{j}\;c_{Y}^{\mathrm{ij}}\;-\frac{2}{ℜ}\int_{ℜ}ds\;\sum_{j=1}^{n}w_{i}\;c_{Y}\;\left(s_{j},s\right) \end{array}$$

which, by condition (b), must be minimized with respect to the weights subject to $\sum_{i=1}^{n}w_{i}=1$, that is, quantity $\sigma_{{\bar{Y}}_{n}^{w}}^{2}+2\theta \;\left[\sum_{i=1}^{n}w_{i}\;-1\right]$ must be minimized with respect to the weights *w_i_* and the Lagrange multiplier *θ*. This leads to the system of equations:

(9) $$\sum_{i=1}^{n}\sum_{j=1}^{n}w_{i}\;w_{j}\;c_{Y}^{\mathrm{ij}}=\frac{1}{ℜ}\int_{ℜ}ds\;\sum_{j=1}^{n}w_{i}\;c_{Y}\;\left(s_{j},s\right)-\sum_{j=1}^{n}w_{i}\;\mu =\frac{1}{ℜ}\int_{ℜ}ds\;\sum_{j=1}^{n}w_{i}\;c_{Y}\;\left(s_{j},s\right)-\theta$$

or in matrix form:

(10) $$\left[\begin{array}{l} c_{Y}\;\left(s_{1},s_{1}\right)\;\;...\;c_{Y}\;\left(s_{1},s_{n}\right)\;\;\;\;1\\ \;\;\;\;\;\;\;\;\;\;\;\;\;\;\;\vdots \\ c_{Y}\;\left(s_{n},s_{1}\right)\;...\;c_{Y}\;\left(s_{n},s_{n}\right)\;1\\ \;\;\;\;\;\;1\;\;\;\;\;\;...\;\;\;\;\;\;\;\;\;1\;\;\;\;\;\;\;0 \end{array}\right]\;\left[\begin{array}{l} w_{i}\\ \;\vdots \\ w_{i}\\ \theta \end{array}\right]=\left[\begin{array}{l} \frac{1}{ℜ}\int_{ℜ}ds\;c_{Y}\;\left(s_{1},s\right)\\ \;\;\;\;\;\;\;\;\;\;\vdots \\ \frac{1}{ℜ}\int_{ℜ}ds\;c_{Y}\;\left(s_{N},s\right)\\ \;\;\;\;\;\;\;\;\;\;1 \end{array}\right]$$

where *C_Y_* denotes the corresponding covariances and *θ* is a Langrange multiplier that accounts for the estimation unbiasedness condition. Note that the Equation (10) above are essentially the block Kriging equations [35] but derived without the assumption of the identical dispersion variance. The integral is evaluated by a summation of the values at regularly discretized points over the area of interest. The integration error is incorporated in the estimation mean variance, see Equation (13) below. The domain boundary effect can be mitigated by drawing more samples around the intersection of the integration grid and the study area boundary.

After the weights *w_i_* and the multiplier *θ* have been calculated from Equation (10), they are substituted back into Equation (4) to obtain the WSM, ${\bar{Y}}_{n}^{w}$. The corresponding minimum error estimation variance of the WSM is given by:

(11) $$\sigma_{{\bar{Y}}_{n}^{w}}^{2}=E{\left[{\bar{Y}}_{n}^{w}-{\bar{Y}}_{ℜ}\right]}^{2}=\frac{1}{{ℜ}^{2}}\int_{ℜ}\int_{ℜ}ds\;ds^{\prime }\;c_{Y}\;\left(s,s^{\prime }\right)\;-\frac{1}{ℜ}\int_{ℜ}ds\;\sum_{j=1}^{n}w_{i}\;c_{Y}\;\left(s,s_{i}\right)-\theta$$

As we shall see below, the set of the mean Kriging Equations (4), (10) and (11) can be implemented with efficiency in the GIS environment.

Let *y* = (*y*_1_,…,*y*_k_) be the random vector (family of random variables) of the SRF *Y*(***s***) at points ***s***_1_,…,***s***_k_. According to probability theory [40], if (*y*_1_, …, *y_n_*) ∼ *N*(***m***, ***V***), then from Equation (4) it is valid that ${\bar{Y}}_{n}^{w}∼N\left(m,\;\sigma^{2}\right)$, where:

(12) $$m=\sum_{i=1}^{n}w_{i}\;E\left[y_{i}\right]$$

[the weights *w_i_* have been calculated from Equation (10)], the assumption of a spatially constant SRF mean still holds, and:

(13) $$\sigma^{2}=\sum_{i=1}^{n}w_{i}^{2}\;\sigma_{y_{i}}^{2}+{2\sum }_{i<j}w_{i}\;w_{j}\;c_{Y}\;\left(s_{i},s_{j}\right)$$

In light of Equation (13), a confident interval of the mean Kriging can be calculated given a confidence level. E.g., with 95% confidence the value of WSM falls into the interval *m* ± 1.96σ. Note that if *y* is shown to be skewed by the Kolmogorov-Smirnov statistics test or it turns out to be non-stationary, then detrending, square root transformation, lognormal transformation etc. may be used to transform ***y*** into a normal probability distribution [40].

In GIS practice to implement the Mean Kriging, one needs to calculate the spatial dependence functions, covariance and variogram, that are related as:

(14) $$\begin{array}{l} c_{Y}^{\mathrm{ij}}=\sigma_{Y}^{2}-\gamma_{Y}^{\mathrm{ij}},\;\;\;\;\;i,j=1,...,N\\ \sigma_{Y}^{2}=c_{0}+c_{1} \end{array}\}$$

where $c_{Y}^{\mathrm{ij}}$ (*i*,*j* = 1,…,*N*) is the covariance between the points ***s****_i_* and ***s****_j_*, $\gamma_{Y}^{\mathrm{ij}}$ is the corresponding variogram, $\sigma_{Y}^{2}$ is the variogram sill, *c*_0_ is the nugget effect and *c*_1_ is the partial sill. Usually, the variogram is first calculated experimentally, then a theoretical model is fitted to the experimental variogram, and finally the corresponding covariance is obtained using Equation (14). To the experimental variogram calculated on the basis of the dataset one can fit one of the available theoretical variogram models [41–44]. E.g., the spherical variogram model is used in the temperature case study considered in this work, see later.

## 5. Case Study I

Next we demonstrate the use of the mean Kriging technique in a GIS environment using a temperature dataset. This dataset includes temperature values (in °C) generated by the remotely sensed image of surface temperature over the study area. We then compare mean area temperature values estimated by simple random sampling, spatial random sampling and ordinary Kriging.

### 5.1. Study Area

The study area is the Shandong Province located in the eastern part of China, along the downstream of the Yellow River and bordering the Bohai Sea and Yellow Sea. Shandong lies in the temperate zone with a half-moisture monsoon climate, an annual average temperature of 12.7 °C and an average annual rainfall of 750 mm. Shandong Province is one of China’s most important agricultural economic regions. The climate change has a significantly impact on the region’s agriculture.

**Figure 1.**
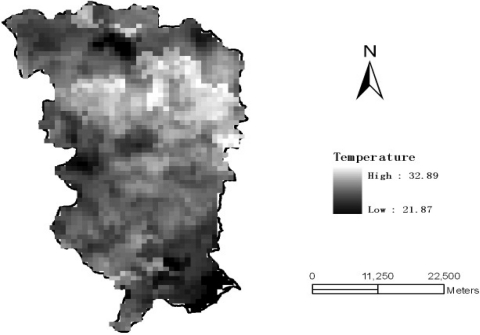
Temperature distribution (in °C), Laiyang county (Shandong, China) at 10:20 pm on May 14^th^, 2007 (MODIS).

**Figure 2.**
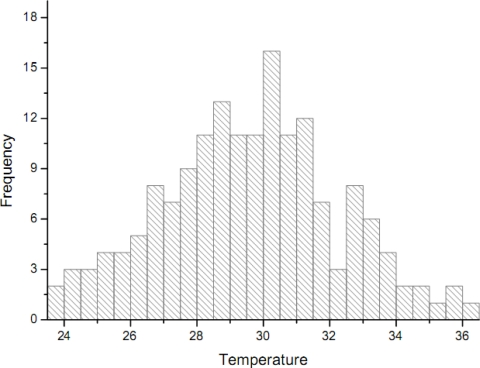
Histogram and simulated pdf of the normal distribution for the temperature dataset (in °C). The dataset belongs to a normal distribution with mean *m* = 26.68 °C and std deviation *σ* = 1.403 °C.

Figure 1 shows the MODIS image of ground temperature in the Laiyang county (Shandong province) obtained at 10:20 pm on May 14^th^, 2007. Each pixel of the MODIS image is regarded as a candidate sample unit. Empirical sample datasets are readily obtained by randomly sampling the image with different proportions The dataset shows that the temperature distribution is very close to the normal distribution (Figure 2). The skew statistics is *S* = 0.038 and the std error is *σ*(*s*) = 0.188 [i.e., S << 2*σ*(*s*)], in which case the skew value indicates that the distribution is almost normal although slightly positively skewed.

### 5.2. Variogram and Covariance Modeling

**Figure 3.**
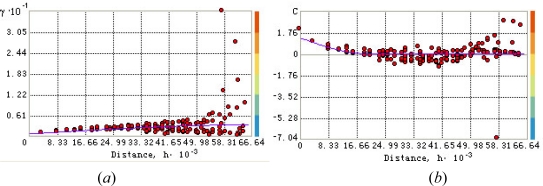
Fitting the spherical model to the experimental: (*a*) variogram and (*b*) covariance temperature values.

Pair-wise correlation (dependence) are calculated using MODIS image of ground surface. To the discrete (experimental) variogram we fitted the spherical variogram model (Figure 3a):

(15) $$\gamma_{Y}^{i\;j}=\left\{ \begin{array}{l} c_{0}+c_{1}\left[1.5\frac{h_{\mathrm{ij}}}{a}-0.5{\left(\frac{h_{\mathrm{ij}}}{a}\right)}^{3}\right]\;\;\text{if}\;h_{\mathrm{ij}}\leq a\\ c_{0}+c_{1}\;\;\text{otherwise} \end{array}\right.$$

where *h_ij_* = ***s****_i_* – ***s****_j_* = *λh* (*h* = 5482.5 *meters* is the lag and *λ* = 1,…,12 is the lag number); *c*_0_ = 0.61848 is the nugget effect, *c*_1_ = 2.667 is the partial sill, and *a* = 64985.6 *meters* is the variogram range, the values are regressed from sample data. The corresponding covariance is as follows (Figure 3b):

(16) $$c_{Y}^{i\;j}=\left\{ \begin{array}{l} c_{1}\left[1-1.5\frac{h_{\mathrm{ij}}}{a}+0.5{\left(\frac{h_{\mathrm{ij}}}{a}\right)}^{3}\right]\;\;\text{if}\;h_{\mathrm{ij}}\leq a\\ 0\;\;\text{otherwise} \end{array}\right.$$

The variogram model (15) was chosen on the basis of experimentation. Several models were tested and the spherical variogram model offered a closer numerical fit to the observed data and also a simpler analytical form (Figure 3). Surely, the present analysis is tailored to the particular dataset of the case study. Hence, one can’t say with certainty that the spherical model offers an ultimate representation of temperature variation. More tests are required to determine a spatial variogram that provides the closest match to regional temperature variation with specified environmental, geophysical and soil characteristics. The maximum dependence range was calculated from the experimental variogram plot. The weighted least square (WLS) technique performed better than the OLS technique in fitting the theoretical model to the experimental variogram; in particular, WLS obtains more accurate spatial continuity estimates than OLS close to the origin (*h* = 0) and it does not need the assumption of normal and independent-identically-distributed (iid) residuals. There is a certain level of model uncertainty in experimental variogram fit, and this has an impact on the mean kriging variance.

### 5.3. Spatial Temperature Mmeans Obtained by the Various Techniques

**Figure 4.**
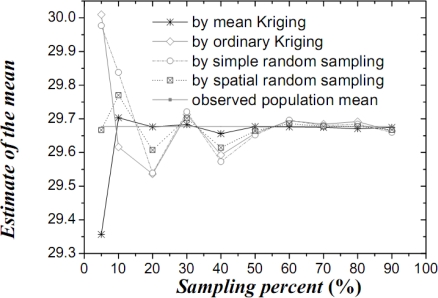
Estimates of the temperature OSPM (in °C) by various techniques.

**Figure 5.**
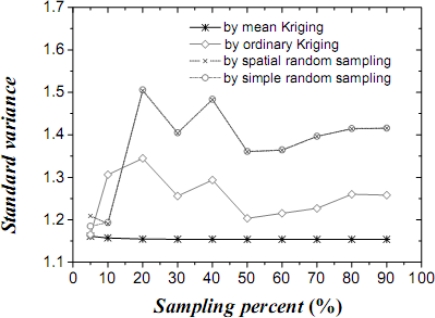
Standard variances of the estimated temperature (in °C) means by the three techniques.

**Figure 6.**
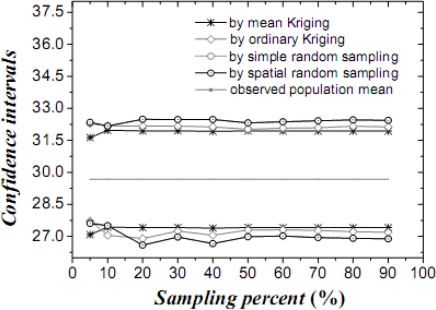
Confidence intervals of the estimated temperature means (in °C) by various techniques.

Table 1 lists the mean values, their variances and confidence intervals using the techniques of simple random sampling, spatial random sampling, ordinary Kriging and mean Kriging, under a sampling proportion of 10%. Figures 4–6 display the means, their standard variances and confidence intervals estimated by the techniques under different sampling proportions.

The results obtained above show that mean Kriging has achieved a better effect, smaller variance and better accuracy of the temperature mean among the proportions ranging from 5% to 90% (5, 10, 20, 30, 40, 50, 60, 70, 80 and 90%). In Figure 4, the temperature mean estimated by mean Kriging is closer to the reference line of the observed temperature value than are the mean values obtained by ordinary Kriging, spatial random sampling and simple random sampling; the sampling proportion varies from 5 to 90%. The relatively small change in “standard variance” with increasing sample size in the case of mean Kriging is linked to the apparently small sill in the modelled variogram (i.e., the data is highly homogeneous so that additional samples add little information about the SRF). Furthermore, Figure 5 shows that the std error variance of spatial mean estimation in terms of mean Kriging technique is minimized, which is not the case with ordinary Kriging, spatial random sampling and simple random sampling. Finally, Figure 6 shows that the confidence intervals obtained by mean Kriging are narrower than other techniques, a fact that indicates the higher accuracy of mean Kriging.

## 6. Case Study II

### 6.1. The Study Region

**Figure 7.**
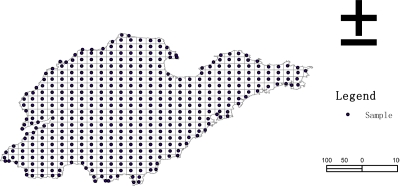
Cultivated land enumerate survey by aerial photos in Shandong (China) in year 2000.

The study region is Shandong province (eastern China). Our aim is to obtain a survey of the proportion of cultivated land in the Shandong province. Actually, the cultivated land and the total territory have already been completely counted in the year 2000 by aerial photos (Figure 7). Table 2 gives a descriptive statistics of the enumerate survey.

### 6.2. Transformation of the Target Variable

**Figure 8.**
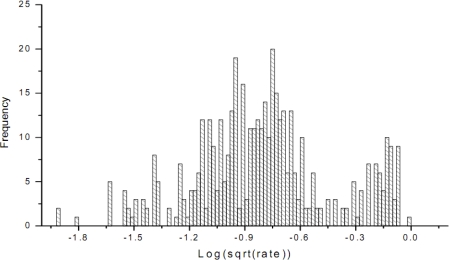
Transformed histogram of crude cultivated land values.

Let the original target variable (the proportion of the cultivated land in Shandong province) be denoted by *x*. The *x* is found to be non-normally distributed, in which case the transform $y=\text{log}(\sqrt{x})$ is conducted. The histogram of the transformed values is shown in Figure 8.

The skew statistics is *S* = 0.09 and the std error is *σ*(*s*) = 0.3722, in which case *S* << 2*σ*(*s*). The skew value indicates that the distribution of the transformed sample attribute is almost normally distributed.

### 6.3. Modeling the Variogram

**Figure 9.**
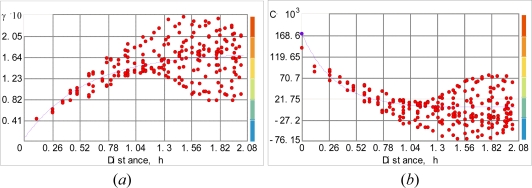
Fitting the spherical model to the experimental: (*a*) variogram and (*b*) covariance.

The experimental variogram and covariance are presented in Figure 9. By exploratory data analysis, we use the spherical variogram model below to simulate the data:

$$\gamma_{Y}^{\mathrm{ij}}=\{\begin{array}{c} 0\;\;\;\;\;\;\;\;\;\;\;\;\;\;\;\;\;\;\;\;\;\;\;\;\;\;\;\;\;\;\;\;\;\;h=0\\ c_{0}+c_{1}\left(1-\text{exp}\left(\frac{{-3h}_{\mathrm{ij}}}{a}\right)\right)\;h>0 \end{array}$$

where the nugget effect is *c*_0_ = 0, the partial sill is *c*_1_ = 0.15775, the major range is *a* = 146.437 *Km; h_ij_* = ***s****_i_* – ***s****_j_* = *λh* (*h* = 33.08 *Km* is the lag and *λ* = 1,…,12 is the lag number);

$$c\left(h_{\mathrm{ij}}\right)=\{\begin{array}{c} 1\;\;\;\;\;\;\;\;\;\;\;\;\;\;\;\;\;\;\;\;\;\;\;\;\;\;\;\;\;\;\;\;\;\;h_{\mathrm{ij}}=0\\ c_{1}\text{exp}\left(\frac{{-3h}_{\mathrm{ij}}}{a}\right)\;h_{\mathrm{ij}}>0 \end{array}$$

### 6.4. Sample Estimates of the Rate of Cultivated Land

**Figure 10.**
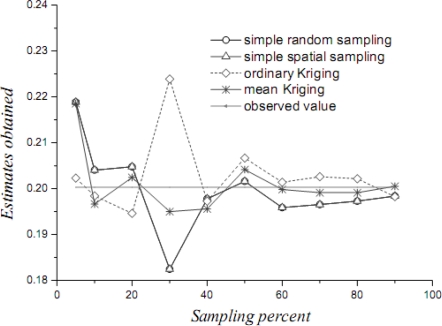
Estimates of the cultivated land OSPM by various techniques.

**Figure 11.**
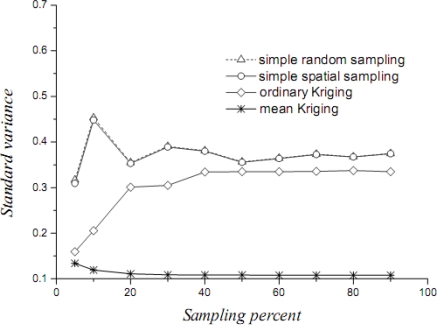
Standard variances of the estimated cultivated land proportion by the three techniques.

**Figure 12.**
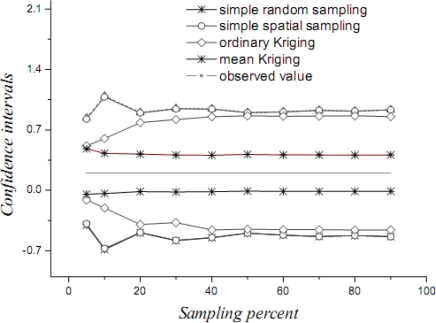
Confidence intervals of the estimated cultivated land proportion by various techniques.

Table 3 presents estimates of the proportion of cultivated land by the various techniques all in 10% sampling proportion; and Figures 10–12 present the parameters, as Figures 4–6.

## 7. Discussion and Conclusions

A method is discussed to estimate the OSPM in a GIS environment. Table 4 summarizes the formulas describing spatial means and the associated variances obtained by different techniques of estimating the OSPM. The estimation variances are ranked as: 0 = variance of observable spatial population mean < variance of mean Kriging mean < variance of ordinary Kriging mean < variance of spatial random sampling mean < variance of simple random sampling mean. The second inequality is due to the fact that ordinary Kriging minimizes the variance at a single site but cannot guarantee minimization of the sample mean variance; the latter is guaranteed by mean Kriging. The comparative advantage of one method over another reduces when the studied area tends to be more homogeneous and less spatially-dependent. In practice, the randomness of empirical cases could lead to insignificant differences.

Although the calculation of a SSM is meaningful, straightforward and unbiased (it has the same expected value as the OSPM and SPM), its variance is not minimized and it suffers from the assumption of equal probability drawing. The OSPM can be estimated by a summation of both values at sampled sites and values at unsampled sites estimated by ordinary Kriging. The Kriging weights attached to different spatial locations within clusters are smaller than those of distanced points, so Kriging is a declustering technique. The mean squared estimation error obtained by kriging was considerably smaller than that of the unweighted sample mean. Although unbiased estimates were derived and their variances were minimized by ordinary Kriging estimation, the spatial mean estimation error (derived by the summation of Kriging values) may accumulate. In addition to the OSPM estimates, the probability distribution of these estimate for the region of interest were derived. These are best linear unbiased OSPM estimates and can be used in more relaxed GIS situations than the original block Kriging.

Using MODIS we generated ground temperature values in the Laiyang county, Shandong Province (China). It was shown that the mean Kriging technique outperformed techniques based on simple random sampling, spatial random sampling and ordinary Kriging in estimating the OSPM of the temperature. The mean Kriging not only accounted for the spatial correlation, as do the conventional spatial sampling techniques, but it also minimized the variance of the objective value as does Kriging. In this study, we focused on the sample estimation and its uncertainty due to sampling design and sample statistics. Since a sample unit is often not uncertainty-free, the sample uncertainty could finally propagate in the context of spatial mean and its variance, which is something that deserves further investigation.

## Figures and Tables

**Table 1.** Spatial temperature means and their confidence intervals estimated by three techniques (10% sampling proportion).

**Technique**	**Spatial mean (°C)**	**Standard deviation (°C)**	**95% confidence interval**
Simple random sampling	29.70	1.194	[27.43, 31.97]
Spatial random sampling	29.84	1.19	[27.50, 32.17]
Ordinary Kriging	29.62	1.31	[27.06, 32.18]
Mean Kriging	29.84	1.16	[27.49, 32.18]

**Table 2.** Descriptive statistics.

N^*^	**Minimum**	**Maximum**	**Mean**	**Std. Deviation**	**Skewness**	
					**Statistic**	**Std. Error**
438	.0218	.9977	.2003	.2171401	1.570	.117

* Note: the observed proportion of cultivated land of Shandong province is 0.265 via completed counting of the coverage.

**Table 3.** Descriptive statistics.

**Technique**	**Spatial mean**	**Standard variance**	**95% confidence interval**
Simple random sampling	0.2040	0.20561	[−0.199, 0.607]
Spatial random sampling	0.2041	0.20083	[−0.199, 0.607]
Ordinary Kriging	0.1984	0.04226	[0.1154, 0.281]
Mean Kriging	0.1966	0.014218	[0.1687, 0.2245]

**Table 4.** Mean and variance formulas of four spatial estimation techniques. The OSPM is also shown.

	**Mean value**	**Variance of mean value**
Simple random sampling [31]	$\frac{1}{N}\sum_{i=1}^{N}y_{i}$	$\begin{array}{c} \frac{1}{N}A\\ \mathrm{where}\;A=\frac{1}{N}{\sum_{i=1}^{N}\left\{y_{i}-E\left[y_{i}\right]\right\}}^{2} \end{array}$
Spatial random sampling [29]	$\frac{1}{N}\sum_{i=1}^{N}y_{i}$	$\begin{array}{c} \frac{1}{N}\left\{A-E\left[c_{Y}\;\left(s_{i},s_{j}\right)\right]\right\}\\ \mathrm{where}\;c_{Y}\;\left(s_{i},s_{j}\right)=\frac{1}{N(N-1)}\sum_{i=1}^{N}\sum_{j=1}^{N-1}\left\{y_{i}-E\left[y_{i}\right]\right\}\left\{y_{j}-E\left[y_{j}\right]\right\})] \end{array}$
Ordinary Kriging [35]	$\sum_{i=1}^{N}w_{i}y_{i}$ and $\frac{1}{N}\sum_{i=1}^{N}y_{i}$	$A-\sum_{i=1}^{N}w_{i}\;E\left[c_{Y}\;\left(s_{i},s_{j}\right)\right]-m$ and $\;\sum_{i=1}^{N}w_{i}\;\sigma_{i}^{2}$
Mean Kriging (this paper)	$\sum_{i=1}^{N}w_{i}\;y_{i}$	$\frac{1}{{ℜ}^{2}}\int_{ℜ}\int_{ℜ}ds\;ds^{\prime }\;c_{Y}\;\left(s,s^{\prime }\right)\;-\frac{1}{ℜ}\int_{ℜ}ds\;\sum_{j=1}^{N}w_{i}\;c_{Y}\;\left(s,s_{i}\right)-m$
Observable spatial population mean (OSPM)	$\frac{1}{ℜ}\int_{ℜ}\mathrm{dsY}(s)$	0
